# An adaptive Bayesian approach for improved sensitivity in joint monitoring of mean and variance using Max-EWMA control chart

**DOI:** 10.1038/s41598-024-60625-2

**Published:** 2024-04-30

**Authors:** Abdullah A. Zaagan, Muhammad Noor-ul-Amin, Imad Khan, Javed Iqbal, Saddam Hussain

**Affiliations:** 1 Department of Mathematics, Faculty of Science, Jazan University, P.O. Box 2097, Jazan, 45142 Kingdom of Saudi Arabia; 2 COMSATS University Islamabad, Lahore Campus, Lahore, Pakistan; 3 Abdul Wali Khan University Mardan, Mardan, Pakistan; 4 ARIA University, Balkh, Afghanistan

**Keywords:** Average run length, Adaptive Max-EWMA, Monte Carlo simulation, Joint monitoring, Control chart, Bayesian approach, Engineering, Mathematics and computing, Physics

## Abstract

This article introduces an adaptive approach within the Bayesian Max-EWMA control chart framework. Various Bayesian loss functions were used to jointly monitor process deviations from the mean and variance of normally distributed processes. Our study proposes the mechanism of using a function-based adaptive method that picks self-adjusting weights incorporated in Bayesian Max-EWMA for the estimation of mean and variance. This adaptive mechanism significantly enhances the effectiveness and sensitivity of the Max-EWMA chart in detecting process shifts in both the mean and dispersion. The Monte Carlo simulation technique was used to calculate the run-length profiles of different combinations. A comparative performance analysis with an existing chart demonstrates its effectiveness. A practical example from the hard-bake process in semiconductor manufacturing is presented for practical context and illustration of the chart settings and performance. The empirical results showcase the superior performance of the Adaptive Bayesian Max-EWMA control chart in identifying out-of-control signals. The chart’s ability to jointly monitor the mean and variance of a process, its adaptive nature, and its Bayesian framework make it a useful and effective control chart.

## Introduction

Statistical process control (SPC) is a field dedicated to maintaining and enhancing the quality of products and processes. It provides a systematic structure that helps in monitoring and controlling the different process variations. Control charts, a basic but powerful tool in SPC, were introduced by Shewhart^1^. Control charts are used to identify the common causes and special causes present in the process. Shewhart control charts are widely used to monitor the shifts in the process mean. However, the Shewhart chart has limitations in detecting minor or gradual process parameter shifts. To address this, the Exponentially Weighted Moving Average (EWMA) chart assigns greater weights to recent data, increasing sensitivity to subtle mean shifts. Conversely, the Cumulative Sum (CUSUM) chart excels at swiftly detecting abrupt changes, responding to both magnitude and direction. These charts were introduced by Page^2^ and Robert^3^. Early control charts focused on single characteristics, but real-world quality standards often demand monitoring of both mean and variance. In practice, processes often exhibit simultaneous variations in mean and variance. Researchers have devised joint monitoring approaches to meet this need. Gan^4^ pioneered a joint monitoring scheme and later Chen and Cheng^5^ introduced the widely adopted Max chart, selecting the maximum absolute value from normalized mean and variance statistics. Chen et al.^6^ incorporated the Max approach with EWMA for joint detection of mean and variance Haq et al.^7^, Sanusi et al.^8^, and Chatterjee et al.^9^ proposed various joint monitoring schemes based on EWMA, CUSUM, and multivariate control charts. Jalilibal et al.^10^ conducted a comprehensive literature review on joint control schemes, highlighting the importance of developing effective and easy-to-use schemes. Joint monitoring schemes for statistical process monitoring remain in the limelight due to their ability to detect changes in both the process mean and dispersion.

The necessity for an adaptive approach in joint monitoring control charts stems from the dynamic nature of contemporary industrial processes. They adjust control limits based on observed data, making them more responsive to process changes. Capizzi and Masarotto^11^ introduced an adaptive approach in EWMA control charts for monitoring processes that exhibit time-varying behavior. Various researchers have worked to make different improvements in adaptive approach. Lee and Lin^12^ offered adaptive Max charts for monitoring the process mean and variability. Huang et al.^13^ evaluate the performance of the adaptive chart with classical charts. Abbas et al.^14^ introduce novel EWMA and CUSUM control charts using sign test statistic and arcsine transformation, demonstrating their performance under in-control and out-of-control processes via Monte Carlo simulation, revealing robustness against non-normality, with sign test statistic effective for small shifts and arcsine transformation for medium to large shifts, applied and assessed using artificial dataset. Abbas et al.^15^ introduce the PAEWMA chart, analyzing its performance under steady-state and zero-state conditions for detecting unknown shifts, comparing it with existing schemes using run-length profiles and quadratic loss measures, highlighting the superior performance of PAEWMA under steady-state conditions, and demonstrating its applicability across artificial, past study, and aircraft accident monitoring datasets. Ugaz et al.^16^ proposed adaptive EWMA charts with time-varying smoothing parameters. Nazir et al.^17^ proposed robust adaptive EWMA charts for manufacturing processes to detect outliers and non-normality. Haq and Razzaq^18^ offered maximum weighted adaptive CUSUM charts for combined monitoring of multiple processes. Sarwar and Noor-ul-Amin^19^ proposed a new adaptive EWMA chart by incorporating a hybrid approach to monitor small and moderate shifts. These studies on adaptive control charts show its acceptance in a wide range of applications. In recent years, the usage of the Bayesian approach in the construction of control charts has drawn significant attention. This is due to the fact that Bayesian methods provide a flexible structure for integrating previous knowledge with the latest available data. Apley^20^ proposed posterior distribution charts for graphically exploring shifts in the process mean. Menzefricke^21^ used a combined EWMA approach based on the predictive distribution to detect shifts. Aunali and Venkatesan^22^ presented a comparative analysis of Bayesian and classical control charts in detecting small shifts. Ali^23^ formulated a predictive Bayesian approach with CUSUM and EWMA charts for monitoring the time between events. Aslam and Anwar^24^ introduced an improved version of the Bayesian Modified-EWMA location chart. Noor-ul-Amin and Noor^25^ developed an adaptive EWMA control chart for monitoring the process mean using different loss functions under the Bayesian approach. Bourazas et al.^26^ proposed predictive control charts (PCCs) using a Bayesian approach for online monitoring of short runs. Khan et al.^27^ implemented various ranked set sampling techniques in Bayesian control charts. These studies demonstrate the versatility of the Bayesian approach in developing control charts. As research on Bayesian control charts continues, it is likely that they will become increasingly common in a wide range of applications.

Bayesian control charts and joint monitoring Max-EWMA control charts are promising approaches for monitoring processes. However, adaptive control charts have not been widely used in conjunction with Max-EWMA joint monitoring. The adaptive weight in adaptive control charts allows them to be more responsive to process shifts. The combination of adaptive control charts and Max-EWMA joint monitoring could lead to the development of more effective control charts for monitoring time-varying processes. In this study, we focus on the comprehensive investigations of these two methodologies with a Bayesian approach to improve the joint monitoring process mean and dispersion.

The remaining article is structured as follows: In section “Bayesian approach”, we cover the Bayesian framework and its various loss functions. In section “Proposed methodology”, the proposed methodology is presented. In section “Major findings and results discussion”, “Real life data application”, and “Conclusion”, we present key findings, comparative study, and practical illustrations of real-life data applications. Finally, section “Conclusion” highlights the conclusion of the study.

## Bayesian approach

There are two main approaches in statistical inference. In the traditional frequentist approach, parameters are considered fixed and unknown. In contrast, the Bayesian approach considers these parameters as probability distributions. In this way, Bayesian theory provides a unique and powerful structure for making inferences based on current and updated observed data. It continuously incorporates prior knowledge to update beliefs as new information emerges. These prior distributions can be broadly categorized into two groups: informative and non-informative. Informative priors depend on a family of distributions and are known as conjugate priors. On the other hand, non-informative priors depend on uniform priors and Jeffrey’s priors. In many fields, the process conditions do not remain fixed but keep changing during the process. Such situations can be better handled with the Bayesian approach. This flexible and intuitive approach is a powerful tool that can be used to develop more effective control charts for a wide range of applications. Let X be a variable of interest with mean $\theta$ and variance $\delta^{2}$. The normal prior distribution with parameters $\theta_{0}$ and $\delta_{0}^{2}$ is mathematical expressed as,

1 $$ p(\theta ) = \frac{1}{{\sqrt {2\pi \delta_{0}^{2} } }}\exp \left\{ { - \frac{1}{{2\delta_{0}^{2} }}\left( {\theta - \theta_{0} } \right)^{2} } \right\} $$

To construct the posterior distribution, we combine the information from the prior distribution and the observed data. This is achieved by setting a proportional relationship through multiplication which combines the likelihood function of the sample distribution with the prior distribution. The resulting posterior distribution

2 $$ p\left( {\theta |x} \right) = \frac{{p\left( {x|\theta } \right) \cdot p\left( \theta \right)}}{{\int {p\left( {x|\theta } \right) \cdot p\left( \theta \right)d\theta } }} $$

To get the updated data values for the posterior predictive (PP) distribution, the posterior distribution serves as the initial reference. Thus, as a fundamental aspect of Bayesian theory, the PP distribution allows for the refinement of prior distributions using fresh data, say *y*. It can be expressed mathematically as,

3 $$ p\left( {y|x} \right) = \int {p\left( {y|\theta } \right) \cdot p\left( {\theta |x} \right)d\theta } $$

### Squared error loss functions

In Bayesian statistics, the SELF quantifies the difference between true parameter values and their estimates, aiding decision-making and parameter estimation. Minimizing this function involves finding estimators that provide close estimates to the true parameter on average. While analytically tractable, it can be sensitive to outliers due to squaring. Nonetheless, it remains a fundamental tool in Bayesian decision theory, guiding the selection of estimators and decision rules balancing bias and variance in estimation and decision-making One such method is the SELF, introduced by Gauss^28^. In this method, we square each value of the estimation error, thereby assigning more weight to larger errors—effectively imposing a greater penalty for greater loss. The ultimate objective is to obtain a posterior mean with a smaller loss. SELF yields robust results, particularly when the posterior follows a normal distribution. Let X be the variable of interest, *θ* is its unknown population parameter and $\hat{\theta }$ is its such estimator that gives minimum loss. So, in this case, SLEF can be equated with the following expression,

4 $$ L\left( {\theta ,\;\hat{\theta }} \right) = \left( {\theta - \hat{\theta }} \right)^{2} $$

The mathematical representation of this estimator that is using Bayes SELF is as follows:

5 $$ \hat{\theta }_{{\left( {SELF} \right)}} = E_{\theta /x} \left( \theta \right) $$

### Linex loss functions

One limitation of using SLEF is that it penalizes all error values, whether they are positive or negative. However, the Linex Loss Function (LLF) treats positive values and negative errors differently. It assigns varying weights to overestimations and underestimations, considering their relative costs. Each outcome may have a different cost, so incorporating its impact in the calculation makes the estimation more efficient. It provides a better way to manage the risk associated with Bayes estimation. This function was introduced by Varian^29^ and is mathematically defined as

6 $$ L\left( {\theta ,\hat{\theta }} \right) = \left( {e^{{c\left( {\theta - \hat{\theta }} \right)}} - c\left( {\theta - \hat{\theta }} \right) - 1} \right) $$

Under LLF, the Bayesian estimator $\hat{\theta }$ becomes as

7 $$ \hat{\theta }_{{\left( {LLF} \right)}} = - \frac{1}{c}InE_{\theta /x} \left( {e^{ - c\theta } } \right) $$

## Proposed methodology

In this section, we present the methodology for incorporating the adaptive approach and Max-EWMA control chart under the Bayesian framework. Here, we assume that the prior distribution is normal. Let *X*_1_, *X*_2_, … *X*_*n*_ be a sequence of normally distributed random variables that are random, independent, and identical. Their mean and variance are denoted by *θ* and $\delta^{2}$. We also consider that the likelihood function is normally distributed. To obtain the posterior distribution, we multiply the prior distribution by the likelihood function. Under these conditions, when both the prior distribution and the likelihood function follow a normal distribution, the resultant posterior distribution will also follow a normal distribution with a mean (*θ*) and variance ($\delta^{2}$). The probability distribution function (pdf) of the posterior distribution in this case can be expressed as

8 $$ P\left( {\theta /x} \right) = \frac{1}{{\sqrt {2\pi } \sqrt {\frac{{\delta^{2} \delta_{0}^{2} }}{{\delta^{2} + n\delta_{0}^{2} }}} }}\exp \left[ { - \frac{1}{2}\left( {\frac{{\theta - \sum\nolimits_{i = 1}^{n} {\frac{{x_{i} \delta_{0}^{2} + \theta_{0} \delta_{0}^{2} }}{{\delta^{2} + n\delta_{0}^{2} }}} }}{{\sqrt {\frac{{\delta^{2} \delta_{0}^{2} }}{{\delta^{2} + n\delta_{0}^{2} }}} }}} \right)^{2} } \right] $$

where $\theta_{n} = \frac{{n\overline{x} \delta_{0}^{2} + \delta^{2} \theta_{0} }}{{\delta^{2} + n\delta_{0}^{2} }}$ and $\delta_{n}^{2} = \frac{{\delta^{2} \delta_{0}^{2} }}{{\delta^{2} + n\delta_{0}^{2} }}$ respectively.

Now we setup Max-EWMA under Bayesian environment. Let's collect different samples of size *n* corresponding to a variable of interest, say *X*, from our underlying process. We standardize these values by applying the following transformation regarding mean and variance. We can transform them using SELF as well as LLF.

### Mean estimation under SELF and LLF

Under SELF, the new transformed expression for calculating the mean is given as

9 $$ U_{t} = \frac{{\hat{\theta }_{(SELF)} - \theta }}{{{\raise0.7ex\hbox{$\delta $} \!\mathord{\left/ {\vphantom {\delta {\sqrt n }}}\right.\kern-0pt} \!\lower0.7ex\hbox{${\sqrt n }$}}}} $$

where $\hat{\theta }_{(SELF)} = \frac{{n\overline{x} \delta_{0}^{2} + \delta^{2} \theta_{0} }}{{\delta^{2} + n\delta_{0}^{2} }}$ is the Bayes estimator for population mean using SELF.

Under LLF, the new transformed expression for calculating mean is expressed as

10 $$ U_{t} = \frac{{\hat{\theta }_{(LLF)} - \theta }}{{{\raise0.7ex\hbox{$\delta $} \!\mathord{\left/ {\vphantom {\delta {\sqrt n }}}\right.\kern-0pt} \!\lower0.7ex\hbox{${\sqrt n }$}}}} $$

where $\hat{\theta }_{{\left( {_{LLF} } \right)}} = \frac{{n\overline{x}_{{(RSS_{i} )}} \delta_{0}^{2} + \delta^{2} \theta_{0} }}{{\delta^{2} + n\delta_{0}^{2} }} - \frac{{C^{\prime}}}{2}\delta_{n}^{2}$ is the Bayes estimator for population mean using LLF.

### Variance estimation under SELF and LLF

Under SLEF, the transformed variance is expressed as,

11 $$ V_{t} = \phi^{ - 1} \left[ {H\left\{ {\frac{{\left( {n - 1} \right)\hat{\delta }_{(SELF)}^{2} }}{{\delta^{2} }}} \right\},\;\;\left( {n - 1} \right)} \right] $$

where $\hat{\delta }_{(SELF)}^{2} = \frac{{\delta^{2} \delta_{0}^{2} }}{{\delta^{2} + n\delta_{0}^{2} }}$ is the Bayes estimators for population variance using SELF.

Under LLF, the transformed expression to estimate the variance becomes

12 $$ V_{t} = \phi^{ - 1} \left[ {H\left\{ {\frac{{\left( {n - 1} \right)\hat{\delta }_{(LLF)}^{2} }}{{\delta^{2} }}} \right\},\left( {n - 1} \right)} \right] $$

where $\hat{\delta }_{{\left( {LLF} \right)}}^{2} = \frac{{\delta^{2} \delta_{0}^{2} }}{{\delta^{2} + n\delta_{0}^{2} }}.$ is the Bayes estimators for population variance using LLF.

In both Eqs. (8) and (9), $H\left( {n,\;\nu } \right)$ follows a chi-square distribution with *v* degrees of freedom and $\phi^{ - 1}$ represents the inverse of the standard normal distribution function.

### Computation of EWMA

We use these transformed values to compute the EWMA statistic for process mean and variance. The expression for EWMA for mean under any LF can be written as

13 $$ P_{t(LF)} = \lambda U_{t(LF)} + \left( {1 - \lambda } \right)P_{t - 1(LF)} $$

and the expression for EWMA for a variance under any LF can be written as

14 $$ Q_{t(LF)} = \lambda V_{t(LF)} + \left( {1 - \lambda } \right)V_{t - 1(LF)} $$

where $P_{0}$ and $Q_{0}$ show the initial values for the EWMA sequences *P*_*t*_ and *Q*_*t*_ respectively and $\lambda$ is chart’s smoothing constant that can be set within the range 0 and 1. When the process is in a stable and in control situation, then both *P*_*t*_ and *Q*_*t*_ are independent and each follows a normal distribution with 0 mean and $\delta_{{P_{t} }}^{2}$ and $\delta_{{Q_{t} }}^{2}$ variances respectively. The simplified expression for variance is

15 $$ \delta_{{P_{t} }}^{2} = \delta_{{Q_{t} }}^{2} = \frac{\lambda }{\lambda - 1}\left[ {1 - \left( {1 - \lambda } \right)^{2t} } \right],\quad {\text{for}}\;\;t = 1,\;2, \ldots $$

### Integration of adaptive approach

Next, we incorporate the adaptive approach. Let ${\text{X}}_{{\text{t}}}$ be a normally distributed random variable, taken at time *t* with a sample of size $n$, with mean as ${\upmu }_{{\text{X}}}$ and variance as ${\upsigma }_{{\text{X}}}^{2}.{\text{ i.e}}.,{{\text{X}}}_{{\text{t}}}\sim {\text{N}}\left({\upmu }_{{\text{X}}}, {\upsigma }_{{\text{X}}}^{2}\right)$.

Jiang et al.^30^ proposed an estimator to estimate the shift size by the following expression

16 $$ \hat{\delta }_{t}^{*} = \psi X_{t} + \left( {1 - \psi } \right)\hat{\delta }_{t - 1}^{*} $$

where $${\uppsi }$$ is the smoothing constant and its range is $$\left( {0,1} \right]$$. In real-world scenarios, it's uncommon to have prior knowledge of the exact magnitude of a shift. To handle this, we first estimate its value. To calculate this Haq et al.^31^ proposed an unbiased estimator, say $$\hat{\delta }_{{\text{t}}}^{{**}}$$, which can be expressed as

17 $$ \hat{\delta }_{t}^{{**}} = \frac{{\hat{\delta }_{t}^{*} }}{{1 - (1 - \psi )^{t} }} $$

where $${\text{E}}\left( {\hat{\delta }_{{\text{t}}}^{{**}} } \right) = {\updelta } = 0$$. The process remains stable for a certain period of time, say t ≤ t_0_. In practical situations, the true magnitude is often unknown. Instead, δ is estimated by considering $${\tilde{\delta }}_{{\text{t}}} = \left| {\hat{\delta }_{{\text{t}}}^{{**}} } \right|$$.

The detection is achieved by recursively calculating the following EWMA statistic, which is referred to as the proposed chart statistic, in the following manner:

18 $$ A_{t} = {\upeta }\left( {{\tilde{\omega }}_{t}^{*} } \right)X_{t} + \left( {1 - {\upeta }\left( {{\tilde{\omega }}_{t}^{*} } \right)} \right)A_{t - 1} $$

where ${\text{A}}_{0}$ = 0 set as the initializing value. Instead of considering fixed value for smoothing constant, here we use a function that can adapt different values according to the process changing conditions. The self-adjusting weighting factor η(${\tilde{\omega }}_{{\text{t}}}^{*}$) is given as

19 $$ \eta \left( {{\tilde{\omega }}_{t}^{*} } \right) = \left\{ {\begin{array}{*{20}c} {\frac{{\tilde{\delta }_{t}^{2} }}{{7\left( {1 + \tilde{\delta }_{t}^{2} } \right)}},} & {\quad \tilde{\delta }_{t} \le 1.0} \\ {\frac{{\tilde{\delta }_{t} }}{{7\left( {1 + \tilde{\delta }_{t} } \right)}},} & {\quad 1.0 < \tilde{\delta }_{t} \le 2.7} \\ {1,} & {\quad \tilde{\delta }_{t} > { }2.7} \\ \end{array} } \right. $$

where *η*($\tilde{\omega }_{t}^{*}$) is a random variable determined by a continuous function, optimizing our chart’s performance for detecting shifts. When $\tilde{\delta }_{t}$ ≤ 2.7, *η*() is tailored for ssitivity to small to moderate shifts. Our adaptive chart excels at detecting shifts of any size, outperforming other methods. If the shift exceeds $\tilde{\delta }_{t}$≥ 2.7, our chart acts like a Shewhart chart, detecting larger shifts with $\tilde{\delta }_{t}$ = 2.7 as the pivot point. It offers flexibility for adjusting the model and incorporating additional factors.

Instead of using fixed $\lambda$ in Eqs. (10) and (11) and assuming that it stays constant throughout the process, we use the self-adapting function of Eq. (16) and recursively update Eqs. (10) and (11) to compute the individual EWMA under adaptive approach. So, the equation becomes,

20 $$ P_{{t\left( {LF} \right)}} = \eta \left( {\tilde{\omega }_{t}^{*} } \right)U_{{t\left( {LF} \right)}} + \left( {1 - \eta \left( {\tilde{\omega }_{t}^{*} } \right)} \right)P_{{t - 1\left( {LF} \right)}} $$

21 $$ Q_{{t\left( {LF} \right)}} = \eta \left( {\tilde{\omega }_{t}^{*} } \right)V_{{t\left( {LF} \right)}} + \left( {1 - \eta \left( {\tilde{\omega }_{t}^{*} } \right)} \right)V_{{t - 1\left( {LF} \right)}} $$

Finally, these values are plugged in Max-EWMA given by Chen and Change^5^ for jointly monitoring the process mean and variance in a single chart. So, the plotting can be expressed as

22 $$ Z_{t} = Max\left( {\left| {P_{t(LF)} } \right|,\;\left| {Q_{t(LF)} } \right|} \right),\quad {\text{for}}\;\;t = 1,\;2, \ldots , $$

where Max is the function to get the maximum value of the given inputs.

As the Adaptive Bayesian Max-EWMA statistic is a positive value, we are required to plot only the upper control limit for jointly monitoring the process mean and variance. The plotting statistic is compared with the UCL threshold. If its value is below UCL then the process is in control. If the value is above the UCL then the process is out of control either by mean, variance, or both.

## Major findings and results discussion

The Tables 1, 2, 3, 4, 5, 6 provides a comprehensive overview of results obtained through the application of the Adaptive Bayesian Max-EWMA control chart method. These evaluations are conducted within the framework of informative priors and based on 50,000 replicates, allowing us to calculate both the ARL and SDRL. We utilized smoothing constants with λ values set at 0.15 and 0.20. Moreover, our study explores a wide range of combinations involving mean shift values (*α*) ranging from 0.00 to 3.00 and variance shift values (*β*) ranging from 1.00 to 3.00. These different combinations are used to measure the performance of the Adaptive Bayesian Max-EWMA control chart method in the monitoring of process mean and variance jointly.

**Table 1:** The run length profiles for proposed Adaptive Bayesian Max-EWMA control chart under SELF, with $\lambda$ = 0.15.

*α*		0.00		0.10		0.25		0.50		0.75		1.00		1.50		2.00		2.50		3.00	
*β*	*n*	ARL	SDRL	ARL	SDRL	ARL	SDRL	ARL	SDRL	ARL	SDRL	ARL	SDRL	ARL	SDRL	ARL	SDRL	ARL	SDRL	ARL	SDRL
1.00	3	370.84	360.47	117.67	87.43	35.35	23.65	11.20	7.14	5.73	3.09	3.73	1.69	2.23	0.82	1.60	0.57	1.23	0.43	1.06	0.23
	5	370.68	368.63	86.75	60.61	23.96	15.56	7.50	4.18	4.03	1.86	2.71	1.17	1.54	0.65	1.11	0.32	1.01	0.10	1.00	0.02
	7	370.56	356.11	70.23	47.82	18.14	11.55	5.83	2.98	3.21	1.47	2.11	0.99	1.20	0.44	1.01	0.11	1.00	0.01	1.00	0.00
1.10	3	91.07	74.17	66.00	51.00	29.27	21.26	10.66	7.18	5.63	3.24	3.75	1.85	2.24	0.89	1.61	0.60	1.26	0.45	1.07	0.26
	5	59.81	44.85	44.99	32.52	19.98	13.85	7.24	4.33	4.04	2.03	2.70	1.26	1.56	0.68	1.13	0.35	1.02	0.13	1.00	0.03
	7	46.02	33.07	35.10	24.60	15.43	10.36	5.66	3.15	3.19	1.57	2.12	1.04	1.23	0.48	1.02	0.15	1.00	0.03	1.00	0.00
1.25	3	26.59	21.16	24.78	19.83	17.79	13.88	9.12	6.56	5.40	3.39	3.69	1.99	2.25	0.98	1.63	0.65	1.29	0.47	1.10	0.30
	5	15.87	11.72	14.95	11.00	11.45	8.16	6.29	3.94	3.83	2.12	2.68	1.36	1.59	0.73	1.17	0.40	1.03	0.17	1.00	0.05
	7	11.60	8.06	11.08	7.69	8.67	5.78	4.95	2.91	3.07	1.66	2.11	1.11	1.27	0.52	1.04	0.19	1.00	0.05	1.00	0.00
1.50	3	9.18	7.38	9.03	7.20	8.15	6.34	6.14	4.48	4.51	2.98	3.41	2.00	2.23	1.10	1.66	0.73	1.33	0.52	1.14	0.36
	5	5.58	3.77	5.51	3.72	5.15	3.41	4.15	2.60	3.18	1.88	2.46	1.37	1.60	0.78	1.21	0.45	1.06	0.24	1.01	0.10
	7	4.22	2.64	4.19	2.61	3.97	2.46	3.24	1.95	2.51	1.46	1.95	1.09	1.29	0.57	1.06	0.25	1.01	0.09	1.00	0.02
2.00	3	3.56	2.47	3.53	2.45	3.45	2.38	3.21	2.16	2.89	1.86	2.56	1.56	2.01	1.10	1.62	0.79	1.36	0.58	1.20	0.43
	5	2.32	1.41	2.30	1.41	2.28	1.39	2.15	1.30	1.98	1.16	1.79	1.01	1.45	0.72	1.21	0.48	1.09	0.30	1.03	0.17
	7	1.80	1.04	1.79	1.04	1.77	1.02	1.68	0.95	1.57	0.85	1.44	0.74	1.21	0.48	1.08	0.29	1.02	0.15	1.00	0.06
2.50	3	2.26	1.41	2.26	1.41	2.25	1.41	2.18	1.35	2.09	1.26	1.97	1.17	1.73	0.95	1.51	0.75	1.34	0.59	1.22	0.46
	5	1.55	0.84	1.55	0.84	1.54	0.83	1.50	0.80	1.46	0.75	1.40	0.70	1.27	0.56	1.16	0.42	1.09	0.30	1.04	0.20
	7	1.26	0.56	1.26	0.56	1.26	0.56	1.24	0.53	1.21	0.50	1.18	0.45	1.11	0.34	1.05	0.24	1.02	0.15	1.01	0.09
3.00	3	1.75	1.01	1.74	0.99	1.74	0.99	1.72	0.97	1.68	0.93	1.63	0.88	1.52	0.78	1.40	0.67	1.30	0.57	1.20	0.45
	5	1.26	0.56	1.26	0.56	1.26	0.55	1.25	0.54	1.23	0.52	1.21	0.49	1.16	0.42	1.11	0.34	1.06	0.26	1.04	0.20
	7	1.10	0.33	1.10	0.33	1.10	0.33	1.10	0.32	1.08	0.30	1.08	0.29	1.05	0.23	1.03	0.18	1.02	0.13	1.01	0.09

**Table 2:** The run length profiles for proposed Adaptive Bayesian Max-EWMA control chart under SELF, with $\lambda$ = 0.20.

α		0.00		0.10		0.25		0.50		0.75		1.00		1.50		2.00		2.50		3.00	
β	*n*	ARL	SDRL	ARL	SDRL	ARL	SDRL	ARL	SDRL	ARL	SDRL	ARL	SDRL	ARL	SDRL	ARL	SDRL	ARL	SDRL	ARL	SDRL
1.00	3	371.76	345.21	122.24	86.52	38.45	23.01	12.98	7.41	6.66	3.31	4.32	1.87	2.23	0.82	1.60	0.57	1.23	0.43	1.06	0.23
	5	370.30	344.98	90.61	60.55	26.54	15.25	8.74	4.48	4.67	2.07	3.05	1.35	1.61	0.76	1.11	0.33	1.01	0.10	1.00	0.01
	7	370.75	366.85	73.71	47.38	20.51	11.58	6.75	3.27	3.66	1.66	2.33	1.15	1.22	0.49	1.01	0.12	1.00	0.01	1.00	0.00
1.10	3	94.39	71.68	69.25	48.96	31.96	20.49	12.24	7.37	6.58	3.51	4.29	2.02	2.24	0.89	1.61	0.60	1.26	0.45	1.07	0.26
	5	63.10	43.36	48.01	31.19	22.30	13.59	8.43	4.63	4.62	2.25	3.04	1.44	1.64	0.80	1.14	0.37	1.02	0.12	1.00	0.03
	7	48.94	32.35	37.81	23.74	17.53	10.39	6.58	3.43	3.62	1.79	2.34	1.21	1.26	0.53	1.02	0.15	1.00	0.03	1.00	0.00
1.25	3	29.35	20.69	27.42	19.20	20.04	13.65	10.57	6.75	6.25	3.59	4.22	2.20	2.25	0.98	1.63	0.65	1.29	0.47	1.10	0.30
	5	17.88	11.73	16.96	10.98	13.18	8.32	7.28	4.25	4.42	2.37	3.00	1.58	1.67	0.85	1.18	0.43	1.03	0.18	1.00	0.05
	7	13.35	8.36	12.73	7.87	10.12	6.06	5.73	3.20	3.48	1.90	2.32	1.28	1.30	0.59	1.04	0.20	1.00	0.05	1.00	0.00
1.50	3	10.62	7.57	10.47	7.42	9.46	6.67	7.20	4.81	5.22	3.25	3.90	2.24	2.23	1.10	1.66	0.73	1.33	0.52	1.14	0.36
	5	6.46	4.12	6.40	4.03	5.95	3.72	4.80	2.92	3.63	2.13	2.73	1.58	1.69	0.91	1.22	0.50	1.06	0.24	1.01	0.10
	7	4.85	2.94	4.79	2.91	4.54	2.76	3.71	2.24	2.82	1.69	2.12	1.26	1.33	0.64	1.07	0.27	1.01	0.09	1.00	0.02
2.00	3	4.04	2.74	4.06	2.79	3.93	2.66	3.65	2.43	3.24	2.10	2.85	1.80	2.01	1.10	1.62	0.79	1.36	0.58	1.20	0.43
	5	2.54	1.63	2.54	1.62	2.50	1.59	2.35	1.49	2.16	1.35	1.92	1.17	1.51	0.83	1.23	0.53	1.09	0.32	1.03	0.17
	7	1.93	1.20	1.91	1.19	1.89	1.17	1.79	1.10	1.65	0.99	1.50	0.85	1.23	0.55	1.08	0.30	1.02	0.15	1.00	0.06
2.50	3	2.47	1.62	2.48	1.63	2.46	1.61	2.38	1.55	2.27	1.45	2.13	1.33	1.73	0.95	1.51	0.75	1.34	0.59	1.22	0.46
	5	1.62	0.95	1.63	0.95	1.61	0.94	1.58	0.91	1.51	0.85	1.45	0.78	1.30	0.63	1.17	0.45	1.09	0.31	1.04	0.21
	7	1.28	0.62	1.29	0.62	1.28	0.61	1.26	0.59	1.23	0.55	1.20	0.50	1.12	0.37	1.05	0.25	1.02	0.15	1.01	0.08
3.00	3	1.85	1.13	1.85	1.14	1.83	1.12	1.81	1.11	1.76	1.06	1.71	1.02	1.52	0.78	1.40	0.67	1.30	0.57	1.20	0.45
	5	1.28	0.61	1.28	0.61	1.28	0.61	1.27	0.59	1.25	0.57	1.23	0.54	1.17	0.46	1.12	0.37	1.07	0.28	1.04	0.21
	7	1.10	0.35	1.11	0.36	1.10	0.35	1.10	0.35	1.09	0.33	1.08	0.30	1.05	0.25	1.03	0.18	1.02	0.13	1.01	0.08

**Table 3:** Comparative of existing Bayesian Max-EWMA control chart and proposed Adaptive Bayesian Max-EWMA control chart under SELF, with $\lambda$ = 0.15.

α			0.00		0.10		0.25		0.50		1.00		2.00		3.00	
β	*n*	Type	ARL	SDRL	ARL	SDRL	ARL	SDRL	ARL	SDRL	ARL	SDRL	ARL	SDRL	ARL	SDRL
1.00	3	Existing	370.0950	365.2474	209.9108	201.1163	54.0980	46.5065	14.1483	8.4748	4.9633	1.7631	2.3219	0.5266	1.7192	0.2783
		Proposed	370.8369	360.4711	117.6749	87.4265	35.3488	23.6503	11.2035	7.1431	3.7337	1.6922	1.5963	0.5744	1.0552	0.2283
	5	Existing	370.2356	364.1423	159.6950	152.5825	32.5425	25.4189	9.2319	4.5814	3.6589	1.0929	1.9308	0.3448	1.1773	0.3819
		Proposed	370.6832	368.6289	86.7464	60.6099	23.9619	15.5557	7.5019	4.1790	2.7061	1.1737	1.1096	0.3161	1.0002	0.0155
	7	Existing	370.2879	363.4935	126.6313	119.0586	23.2994	16.7468	7.1665	3.1000	3.0334	0.8171	1.6825	0.4681	1.0150	0.1215
		Proposed	370.5644	356.1078	70.2331	47.8194	18.1380	11.5507	5.8326	2.9831	2.1104	0.9869	1.0132	0.1144	1.0000	0.0000
1.50	3	Existing	11.9084	7.8667	11.6904	7.6825	10.5982	6.6942	8.2143	4.6934	4.7135	2.1296	2.3871	0.7419	1.6709	0.5207
		Proposed	9.1807	7.3751	9.0332	7.2040	8.1454	6.3377	6.1360	4.4775	3.4105	2.0014	1.6575	0.7279	1.1429	0.3575
	5	Existing	7.3073	3.9265	7.2076	3.8278	6.8140	3.4989	5.6582	2.6500	3.5234	1.3296	1.9076	0.5443	1.2691	0.4441
		Proposed	5.5847	3.7696	5.5130	3.7158	5.1509	3.4086	4.1497	2.5970	2.4595	1.3684	1.2074	0.4499	1.0100	0.0995
	7	Existing	5.5734	2.6158	5.5318	2.5680	5.2939	2.3824	4.5478	1.8702	2.9553	1.0103	1.6433	0.5167	1.0735	0.2609
		Proposed	4.2187	2.6401	4.1949	2.6142	3.9712	2.4645	3.2431	1.9485	1.9479	1.0877	1.0633	0.2530	1.0004	0.0205
2.00	3	Existing	5.0767	2.6626	5.0556	2.6606	4.9461	2.5796	4.6197	2.3474	3.7112	1.7207	2.3528	0.8943	1.6745	0.5974
		Proposed	3.5588	2.4692	3.5263	2.4468	3.4498	2.3755	3.2142	2.1626	2.5628	1.5582	1.6160	0.7879	1.1988	0.4291
	5	Existing	3.3971	1.4363	3.3972	1.4320	3.3553	1.4030	3.2148	1.3167	2.7468	1.0419	1.8542	0.6364	1.3135	0.4716
		Proposed	2.3204	1.4087	2.3011	1.4066	2.2769	1.3934	2.1546	1.2987	1.7892	1.0081	1.2139	0.4774	1.0295	0.1720
	7	Existing	2.7436	1.0182	2.7401	1.0246	2.7204	1.0088	2.6335	0.9469	2.3144	0.7915	1.5984	0.5548	1.1361	0.3435
		Proposed	1.8000	1.0449	1.7945	1.0396	1.7687	1.0230	1.6804	0.9546	1.4413	0.7382	1.0789	0.2870	1.0038	0.0617
3.00	3	Existing	2.5667	1.2163	2.5594	1.2097	2.5406	1.2033	2.5079	1.1894	2.3680	1.0969	1.9643	0.8628	1.5976	0.6626
		Proposed	1.7506	1.0054	1.7405	0.9905	1.7399	0.9877	1.7154	0.9717	1.6284	0.8836	1.4004	0.6686	1.2007	0.4519
	5	Existing	1.8523	0.7359	1.8546	0.7384	1.8492	0.7393	1.8380	0.7327	1.7579	0.6991	1.5210	0.5905	1.2842	0.4692
		Proposed	1.2649	0.5563	1.2634	0.5562	1.2603	0.5484	1.2510	0.5390	1.2102	0.4885	1.1103	0.3437	1.0390	0.1995
	7	Existing	1.5460	0.5870	1.5515	0.5906	1.5423	0.5871	1.5313	0.5831	1.4792	0.5610	1.3027	0.4759	1.1268	0.3343
		Proposed	1.1015	0.3336	1.0989	0.3285	1.0961	0.3256	1.0954	0.3244	1.0766	0.2882	1.0299	0.1765	1.0071	0.0856

**Table 4:** The run length profiles for proposed Adaptive Bayesian Max-EWMA control chart under LLF, with $\lambda$ = 0.15.

*Α*		0.00		0.10		0.25		0.50		0.75		1.00		1.50		2.00		2.50		3.00	
*β*	*N*	ARL	SDRL	ARL	SDRL	ARL	SDRL	ARL	SDRL	ARL	SDRL	ARL	SDRL	ARL	SDRL	ARL	SDRL	ARL	SDRL	ARL	SDRL
1.00	3	370.59	360.78	118.26	88.40	35.88	23.96	11.29	7.15	5.75	3.10	3.74	1.69	2.23	0.82	1.60	0.57	1.24	0.43	1.06	0.23
	5	370.01	358.53	87.63	61.56	24.09	15.66	7.51	4.17	4.04	1.87	2.71	1.17	1.54	0.65	1.11	0.32	1.01	0.09	1.00	0.02
	7	370.42	359.58	70.60	47.96	18.29	11.62	5.83	2.98	3.22	1.46	2.12	0.99	1.20	0.43	1.01	0.11	1.00	0.01	1.00	0.00
1.10	3	91.32	74.70	66.54	50.95	29.70	21.78	10.70	7.23	5.69	3.28	3.74	1.82	2.23	0.89	1.61	0.60	1.26	0.45	1.07	0.26
	5	60.36	45.13	45.31	32.83	19.90	13.80	7.27	4.35	4.00	2.01	2.71	1.26	1.56	0.68	1.14	0.35	1.02	0.12	1.00	0.03
	7	46.21	33.10	35.31	24.88	15.53	10.42	5.70	3.14	3.20	1.59	2.12	1.04	1.23	0.47	1.02	0.15	1.00	0.03	1.00	0.00
1.25	3	27.00	21.59	25.12	20.02	17.89	14.00	9.16	6.58	5.38	3.33	3.68	1.97	2.25	0.99	1.63	0.65	1.29	0.47	1.10	0.30
	5	15.88	11.74	15.05	11.09	11.56	8.21	6.26	3.96	3.86	2.12	2.68	1.37	1.60	0.74	1.17	0.39	1.03	0.17	1.00	0.05
	7	11.67	8.17	11.08	7.72	8.70	5.76	4.94	2.92	3.07	1.67	2.12	1.11	1.27	0.52	1.04	0.20	1.00	0.04	1.00	0.01
1.50	3	9.23	7.36	9.05	7.17	8.14	6.37	6.17	4.50	4.51	2.96	3.41	1.99	2.23	1.11	1.65	0.72	1.33	0.52	1.14	0.36
	5	5.60	3.79	5.54	3.74	5.16	3.42	4.13	2.59	3.18	1.87	2.45	1.37	1.60	0.79	1.21	0.45	1.06	0.23	1.01	0.10
	7	4.22	2.63	4.18	2.62	3.94	2.43	3.27	1.95	2.53	1.46	1.94	1.09	1.29	0.56	1.06	0.25	1.01	0.09	1.00	0.02
2.00	3	3.55	2.46	3.56	2.46	3.43	2.36	3.22	2.16	2.91	1.88	2.58	1.58	2.01	1.09	1.62	0.79	1.37	0.59	1.20	0.44
	5	2.32	1.42	2.30	1.41	2.28	1.40	2.16	1.29	1.99	1.17	1.78	1.01	1.45	0.72	1.22	0.48	1.09	0.30	1.03	0.17
	7	1.81	1.05	1.78	1.03	1.77	1.03	1.69	0.96	1.57	0.86	1.44	0.73	1.21	0.49	1.08	0.28	1.02	0.14	1.00	0.06
2.50	3	2.27	1.42	2.28	1.44	2.25	1.42	2.18	1.34	2.09	1.27	1.97	1.16	1.73	0.94	1.51	0.75	1.35	0.59	1.22	0.46
	5	1.55	0.84	1.54	0.84	1.54	0.83	1.51	0.80	1.45	0.75	1.40	0.70	1.27	0.56	1.16	0.42	1.08	0.29	1.04	0.20
	7	1.27	0.57	1.26	0.56	1.26	0.55	1.24	0.53	1.21	0.49	1.18	0.45	1.11	0.35	1.05	0.24	1.02	0.15	1.01	0.09
3.00	3	1.75	1.01	1.75	1.00	1.74	0.99	1.73	0.97	1.68	0.94	1.63	0.89	1.52	0.79	1.40	0.67	1.29	0.55	1.20	0.45
	5	1.27	0.56	1.26	0.56	1.26	0.55	1.24	0.53	1.23	0.51	1.21	0.48	1.16	0.42	1.11	0.34	1.07	0.26	1.04	0.20
	7	1.10	0.33	1.10	0.33	1.10	0.33	1.09	0.32	1.08	0.30	1.07	0.28	1.05	0.23	1.03	0.18	1.02	0.12	1.01	0.08

**Table 5:** The run length profiles for proposed Adaptive Bayesian Max-EWMA control chart under LLF, with $\lambda$ = 0.20.

α		0.00		0.10		0.25		0.50		0.75		1.00		1.50		2.00		2.50		3.00	
β	*n*	ARL	SDRL	ARL	SDRL	ARL	SDRL	ARL	SDRL	ARL	SDRL	ARL	SDRL	ARL	SDRL	ARL	SDRL	ARL	SDRL	ARL	SDRL
1.00	3	370.73	344.19	123.23	87.77	38.77	22.91	13.05	7.39	6.69	3.34	4.30	1.87	2.47	0.97	1.66	0.66	1.24	0.44	1.06	0.23
	5	370.41	343.43	91.40	61.13	26.49	15.18	8.70	4.47	4.66	2.09	3.06	1.35	1.62	0.76	1.11	0.33	1.01	0.09	1.00	0.02
	7	370.27	344.98	74.67	47.76	20.46	11.52	6.75	3.26	3.65	1.66	2.34	1.15	1.22	0.49	1.01	0.12	1.00	0.01	1.00	0.00
1.10	3	95.70	73.16	69.38	48.80	32.31	20.76	12.30	7.41	6.59	3.52	4.29	2.01	2.47	1.04	1.67	0.70	1.27	0.47	1.07	0.26
	5	63.58	44.04	48.42	31.54	22.36	13.78	8.41	4.61	4.63	2.24	3.04	1.44	1.65	0.80	1.14	0.37	1.02	0.13	1.00	0.03
	7	48.74	31.82	37.95	23.91	17.49	10.47	6.59	3.44	3.61	1.80	2.34	1.21	1.26	0.53	1.02	0.15	1.00	0.03	1.00	0.00
1.25	3	29.52	20.66	27.57	19.30	20.22	13.73	10.61	6.80	6.24	3.62	4.25	2.20	2.48	1.15	1.71	0.75	1.30	0.50	1.10	0.31
	5	18.11	11.87	17.07	11.10	13.23	8.38	7.28	4.22	4.44	2.39	2.99	1.56	1.67	0.85	1.18	0.43	1.03	0.17	1.00	0.05
	7	13.31	8.35	12.73	7.95	10.20	6.17	5.79	3.22	3.50	1.90	2.32	1.29	1.30	0.59	1.04	0.20	1.00	0.05	1.00	0.01
1.50	3	10.63	7.57	10.41	7.40	9.55	6.72	7.20	4.81	5.26	3.25	3.89	2.24	2.43	1.27	1.74	0.84	1.35	0.56	1.15	0.37
	5	6.47	4.10	6.40	4.06	6.01	3.75	4.81	2.94	3.63	2.13	2.72	1.58	1.69	0.91	1.23	0.50	1.06	0.24	1.01	0.10
	7	4.84	2.96	4.78	2.90	4.53	2.74	3.71	2.24	2.81	1.70	2.10	1.26	1.33	0.64	1.07	0.27	1.01	0.10	1.00	0.02
2.00	3	4.04	2.76	4.01	2.74	3.93	2.66	3.66	2.43	3.26	2.12	2.86	1.79	2.17	1.27	1.69	0.90	1.39	0.64	1.21	0.46
	5	2.56	1.64	2.55	1.62	2.51	1.60	2.35	1.49	2.16	1.35	1.93	1.17	1.51	0.83	1.24	0.53	1.09	0.32	1.03	0.17
	7	1.92	1.20	1.92	1.20	1.88	1.17	1.79	1.10	1.65	0.98	1.50	0.85	1.23	0.56	1.08	0.30	1.02	0.15	1.00	0.06
2.50	3	2.47	1.63	2.48	1.62	2.44	1.61	2.37	1.55	2.26	1.46	2.13	1.33	1.83	1.09	1.57	0.84	1.37	0.65	1.23	0.50
	5	1.62	0.95	1.63	0.97	1.62	0.94	1.57	0.91	1.51	0.85	1.45	0.78	1.30	0.62	1.17	0.46	1.09	0.31	1.04	0.21
	7	1.29	0.62	1.29	0.62	1.28	0.62	1.26	0.59	1.23	0.55	1.19	0.50	1.11	0.37	1.06	0.25	1.02	0.16	1.01	0.09
3.00	3	1.85	1.15	1.84	1.13	1.85	1.14	1.80	1.10	1.77	1.07	1.71	1.01	1.58	0.89	1.44	0.75	1.32	0.62	1.22	0.49
	5	1.29	0.61	1.28	0.60	1.28	0.60	1.27	0.59	1.25	0.56	1.23	0.54	1.17	0.46	1.12	0.37	1.07	0.27	1.04	0.20
	7	1.11	0.36	1.10	0.35	1.10	0.35	1.10	0.34	1.09	0.33	1.08	0.31	1.05	0.25	1.03	0.19	1.02	0.13	1.01	0.09

**Table 6:** Comparative of existing Bayesian Max-EWMA control chart and proposed Adaptive Bayesian Max-EWMA control chart under LLF, with $\lambda$ = 0.15.

α			0.00		0.10		0.25		0.50		1.00		2.00		3.00	
β	*n*	Type	ARL	SDRL	ARL	SDRL	ARL	SDRL	ARL	SDRL	ARL	SDRL	ARL	SDRL	ARL	SDRL
1.00	3	Existing	370.4812	361.5483	213.5145	207.2202	53.9520	46.5927	14.1522	8.5403	5.0008	1.7912	2.3231	0.5262	1.7197	0.4528
		Proposed	370.5927	360.7761	118.2624	88.3971	35.8776	23.9603	11.2896	7.1516	3.7371	1.6875	1.5999	0.5729	1.0564	0.2306
	5	Existing	370.9033	363.5844	162.2798	155.7887	32.6641	25.5111	9.2280	4.6530	3.6607	1.0896	1.9327	0.3550	1.1746	0.3796
		Proposed	370.0055	358.5289	87.6300	61.5614	24.0901	15.6633	7.5067	4.1691	2.7087	1.1714	1.1083	0.3154	1.0003	0.0173
	7	Existing	371.0654	363.2578	128.0419	121.3617	23.2918	16.7425	7.1525	3.1274	3.0423	0.8174	1.6842	0.4678	1.0153	0.1228
		Proposed	370.4160	359.5801	70.6050	47.9559	18.2950	11.6167	5.8302	2.9805	2.1209	0.9878	1.0122	0.1103	1.0000	0.0000
1.50	3	Existing	11.9084	7.8667	11.6976	7.6790	10.7185	6.7715	8.2049	4.6471	4.7185	2.1228	2.3869	0.7457	1.6742	0.5214
		Proposed	9.2276	7.3577	9.0460	7.1729	8.1424	6.3706	6.1683	4.5017	3.4070	1.9924	1.6534	0.7212	1.1435	0.3574
	5	Existing	7.3047	3.9346	7.2358	3.8663	6.8104	3.4807	5.6520	2.6654	3.5255	1.3368	1.9086	0.5484	1.2662	0.4432
		Proposed	5.5979	3.7899	5.5431	3.7432	5.1626	3.4193	4.1315	2.5869	2.4478	1.3655	1.2089	0.4518	1.0095	0.0969
	7	Existing	5.5863	2.6044	5.5510	2.5868	5.3417	2.4228	4.5315	1.8537	2.9523	1.0035	1.6423	0.5186	1.0756	0.2644
		Proposed	4.2188	2.6252	4.1830	2.6208	3.9406	2.4323	3.2672	1.9489	1.9445	1.0916	1.0637	0.2538	1.0005	0.0224
2.00	3	Existing	5.0767	2.6626	5.0710	2.6698	4.9540	2.5825	4.6364	2.3392	3.7137	1.7244	2.3450	0.8923	1.6761	0.5999
		Proposed	3.5525	2.4581	3.5591	2.4581	3.4290	2.3552	3.2244	2.1574	2.5798	1.5764	1.6157	0.7864	1.2028	0.4353
	5	Existing	3.4137	1.4501	3.4130	1.4340	3.3401	1.3977	3.2172	1.3205	2.7518	1.0511	1.8557	0.6377	1.3159	0.4740
		Proposed	2.3223	1.4208	2.3013	1.4083	2.2798	1.3978	2.1561	1.2853	1.7841	1.0120	1.2172	0.4815	1.0287	0.1692
	7	Existing	2.7425	1.0242	2.7284	1.0176	2.7263	1.0107	2.6271	0.9479	2.3193	0.7919	1.5943	0.5572	1.1317	0.3387
		Proposed	1.8051	1.0479	1.7784	1.0329	1.7678	1.0250	1.6861	0.9570	1.4368	0.7336	1.0773	0.2839	1.0041	0.0641
3.00	3	Existing	2.5667	1.2163	2.5583	1.2064	2.5478	1.2066	2.5123	1.1816	2.3849	1.1054	1.9733	0.8731	1.5931	0.6591
		Proposed	1.7538	1.0069	1.7511	1.0050	1.7417	0.9920	1.7289	0.9720	1.6283	0.8858	1.4004	0.6694	1.2019	0.4539
	5	Existing	1.8574	0.7435	1.8528	0.7384	1.8520	0.7445	1.8295	0.7303	1.7597	0.6923	1.5212	0.5947	1.2759	0.4650
		Proposed	1.2655	0.5554	1.2646	0.5559	1.2611	0.5543	1.2422	0.5272	1.2076	0.4829	1.1088	0.3418	1.0386	0.1980
	7	Existing	1.5477	0.5877	1.5484	0.5897	1.5420	0.5880	1.5288	0.5785	1.4786	0.5604	1.3011	0.4733	1.1245	0.3320
		Proposed	1.1023	0.3330	1.1013	0.3331	1.0984	0.3292	1.0927	0.3183	1.0727	0.2820	1.0325	0.1836	1.0063	

### Effect of mean shift on ARL and SDRL

As the mean is shifted from 0.00 to 3.00, there is a noticeable trend in both the ARL and SDRL. ARL values decrease considerably with the higher mean shifts. These results show that the method becomes more sensitive to shifts in data as the mean shift increases. On the other hand, SDRL is decreased with the increase in mean shifts. This behavior of SDRL is aligned with ARL observations. The performance of the chart becomes more consistent and less variable, which is a desirable characteristic in real-world applications where stable and reliable change detection is essential.

### Effect of variance shift on ARL and SDRL

Similar to the mean shift, variance shift exhibits a consistent influence on ARL and SDRL as it increases from 1.00 to 3.00. ARL decreases with higher v shift values, indicating that the algorithm becomes more efficient at detecting changes when variance shift is increased. This result aligns with the observed behavior of mean shift and suggests that a higher v shift can lead to faster change detection. Correspondingly, the decrease in SDRL as variance shift increases implies that the algorithm's detection performance becomes more stable and predictable. This predictability is valuable in applications where consistent and reliable change detection is paramount.

### Effect of sample size on ARL and SDRL

The sample size (*n*) in the table has a noticeable impact on the results. Larger sample sizes (e.g., *n* = 7) consistently lead to quicker and more stable change detection, as evidenced by lower ARL and SDRL values. This increased responsiveness and reliability in detecting changes make larger sample sizes a favorable choice in practical applications where timely and consistent detection is crucial. However, it's essential to consider computational resources and time constraints when selecting the sample size, as larger samples may come with increased processing demands.

### Effect of smoothing constant on ARL and SDRL

The different values of the smoothing constant were also compared, specifically λ = 0.15 and λ = 0.20. We can see notable differences in the ARL and SDRL values. For λ = 0.15, the adaptive Bayesian Max-EWMA control chart method generally exhibits higher ARL values across varying mean shift and variance shift scenarios. In contrast, with λ = 0.20, the Adaptive method consistently achieves lower ARL values, indicating faster change detection. This observation suggests that a higher λ value enhances the chart's sensitivity which leads to quicker detection.

The results from Tables 4, 5, 6 are computed under the LLF. The analysis of the ARL and SDRL under LLF also shows the similar observation as found in Table 1, 2, 3 under SELF. We can say that our proposed control chart performs equally well under both loss functions namely SELF and LLF.

### Comparative study

In this study, we conduct a comparative analysis of the two control charts: the Bayesian Max-EWMA chart and our proposed Adaptive Bayesian Max-EWMA chart. Control charts are vital tools for quality control and process monitoring. The Bayesian Max-EWMA chart is a well-established method, while our Adaptive Bayesian Max-EWMA chart is integrated with an adaptive approach to changing process conditions. The examination of ARL and SDRL values under various scenarios provides insights into the performance of these charts. Following are the findings from the results.

i. As the mean shift increases the ARL and SDRL values of both charts get decrease. However, the decrease in ARL and SDRL values is more prominent in the proposed Adaptive chart. This shows its higher sensitivity to detect changes in the process.
ii. As the variance shift increases, it generally leads to higher ARL values for both charts. The proposed Adaptive chart consistently shows better performance than the counterpart chart. It shows lower ARL values across different shift levels of variance.
iii. Overall, the proposed Adaptive Bayesian Max-EWMA chart consistently has lower ARL values compared to the Bayesian Max-EWMA chart. In most cases, the SDRL values for the Adaptive chart are also lower than those for the counterpart chart. This show stability in the repeated results. The Adaptive chart consistently performs better in terms of faster detection and lower variability in run length.

## Real life data application

In this section, we apply the proposed Adaptive Bayesian Max-EWMA chart to real-life data and compare its performance with the existing Bayesian Max-EWMA chart. In semiconductor manufacturing, integrated circuits (ICs) are placed on a thin slice-like material called a wafer. The flow width of the photoresist is a critical dimension that must be controlled to ensure the proper functioning of the ICs. By monitoring the flow width of the photoresist, engineers can ensure that the process is producing high-quality results. Montgomery^32^ provides an example of a hard baking process in semiconductor manufacturing. The dataset consists of 45 samples, each with 5 values, and each sample is collected every hour. The first 30 values serve as in-control values, while the last 15 values are contaminated with impurities. Both charts are used to monitor the process variations in the mean and variance, and the computed results are presented in Table 7. In this demonstration, we have used SELF only.

**Table 7:** The values and out of control status Bayesian Max-EWMA control chart and proposed Adaptive Bayesian Max-EWMA under SELF, with $\lambda$ = 0.20. Significant values are in bold.

Sample #	Phase I and II	Bayesian Max-EWMA	UCL	Out of Control (ooc) Status	Proposed Adaptive Bayesian Max-EWMA	UCL	Out of Control (ooc) Status
1	Phase I	0.6972	4.3309	0	3.8979	5.3681	0
2	Phase I	1.4723	4.3309	0	3.5484	5.3681	0
3	Phase I	2.0851	4.3309	0	3.5131	5.3681	0
4	Phase I	2.3224	4.3309	0	4.8416	5.3681	0
5	Phase I	2.6040	4.3309	0	4.8638	5.3681	0
6	Phase I	2.7582	4.3309	0	4.2898	5.3681	0
7	Phase I	2.8424	4.3309	0	4.1213	5.3681	0
8	Phase I	3.0395	4.3309	0	3.5611	5.3681	0
9	Phase I	3.1608	4.3309	0	3.6640	5.3681	0
10	Phase I	3.2400	4.3309	0	4.4031	5.3681	0
11	Phase I	3.2875	4.3309	0	3.4912	5.3681	0
12	Phase I	3.3622	4.3309	0	4.3393	5.3681	0
13	Phase I	3.3487	4.3309	0	3.1377	5.3681	0
14	Phase I	3.4667	4.3309	0	3.8406	5.3681	0
15	Phase I	3.5468	4.3309	0	4.0582	5.3681	0
16	Phase I	3.5445	4.3309	0	3.9459	5.3681	0
17	Phase I	3.7963	4.3309	0	3.2276	5.3681	0
18	Phase I	3.7679	4.3309	0	5.0247	5.3681	0
19	Phase I	3.7366	4.3309	0	4.3252	5.3681	0
20	Phase I	3.8253	4.3309	0	4.6634	5.3681	0
21	Phase I	3.9060	4.3309	0	3.3422	5.3681	0
22	Phase I	3.8549	4.3309	0	3.5624	5.3681	0
23	Phase I	3.7873	4.3309	0	3.6537	5.3681	0
24	Phase I	3.7462	4.3309	0	3.3803	5.3681	0
25	Phase I	3.6215	4.3309	0	3.6562	5.3681	0
26	Phase I	3.5263	4.3309	0	3.8468	5.3681	0
27	Phase I	3.7080	4.3309	0	3.8858	5.3681	0
28	Phase I	3.7837	4.3309	0	3.9581	5.3681	0
29	Phase I	3.7975	4.3309	0	3.4737	5.3681	0
30	Phase I	3.9149	4.3309	0	3.4326	5.3681	0
31	Phase II	3.8868	4.3309	0	3.5970	5.3681	0
32	Phase II	3.8659	4.3309	0	3.3344	5.3681	0
33	Phase II	3.7842	4.3309	0	3.3788	5.3681	0
34	Phase II	3.7226	4.3309	0	3.5064	5.3681	0
35	Phase II	3.6624	4.3309	0	3.0590	5.3681	0
36	Phase II	3.6668	4.3309	0	3.6089	5.3681	0
37	Phase II	3.7162	4.3309	0	5.6047	5.3681	1
38	Phase II	3.7841	4.3309	0	6.3014	5.3681	1
39	Phase II	4.3023	4.3309	0	6.8634	5.3681	1
40	Phase II	4.8939	4.3309	1	7.4433	5.3681	1
41	Phase II	5.4813	4.3309	1	7.9585	5.3681	1
42	Phase II	6.0585	4.3309	1	8.4963	5.3681	1
43	Phase II	6.6211	4.3309	1	9.0114	5.3681	1
44	Phase II	7.1640	4.3309	1	9.4912	5.3681	1
45	Phase II	7.6870	4.3309	1	9.9170	5.3681	1

**Figure 1:**
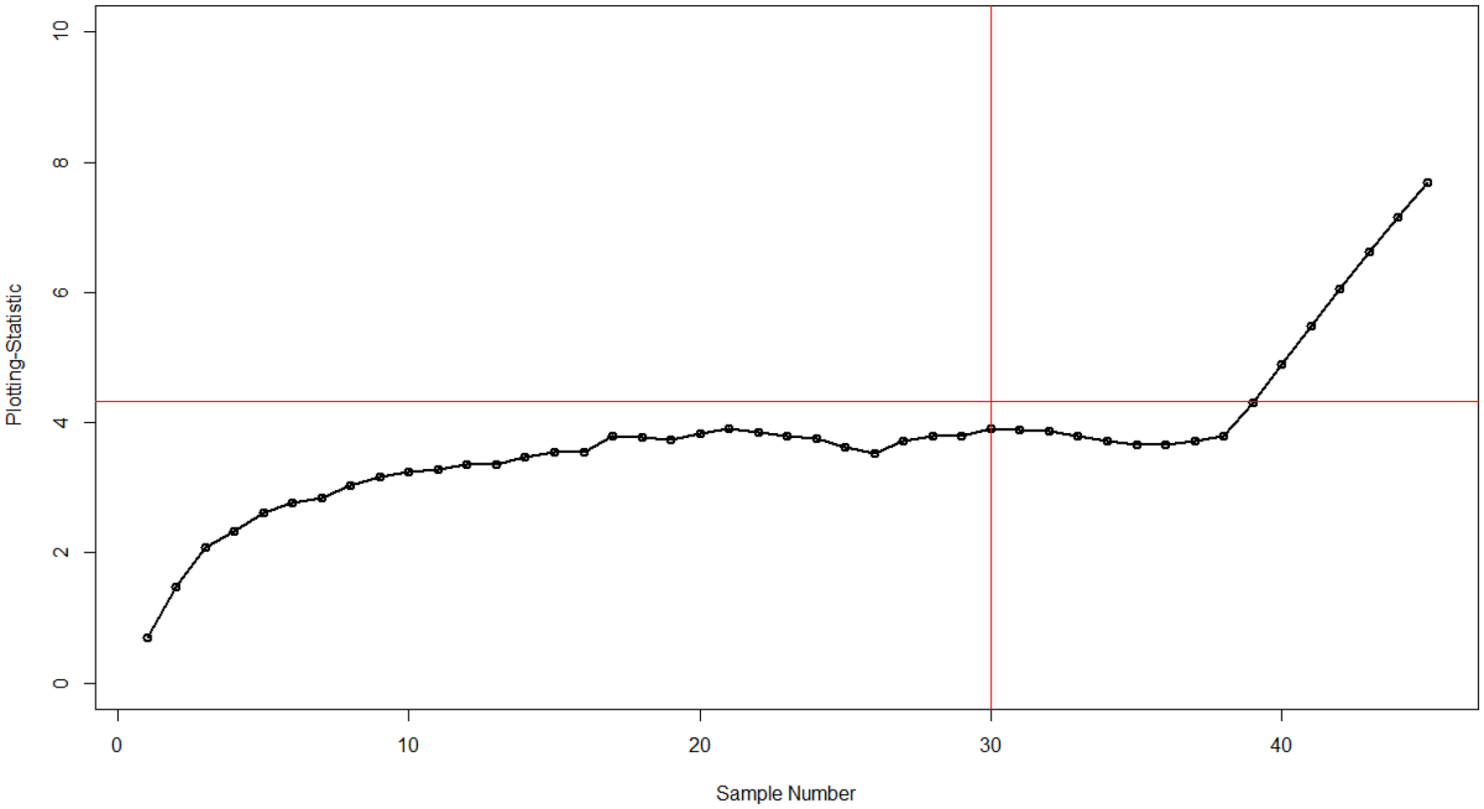
Bayesian Max-EWMA under SELF with $\lambda$ = 0.20.

**Figure 2:**
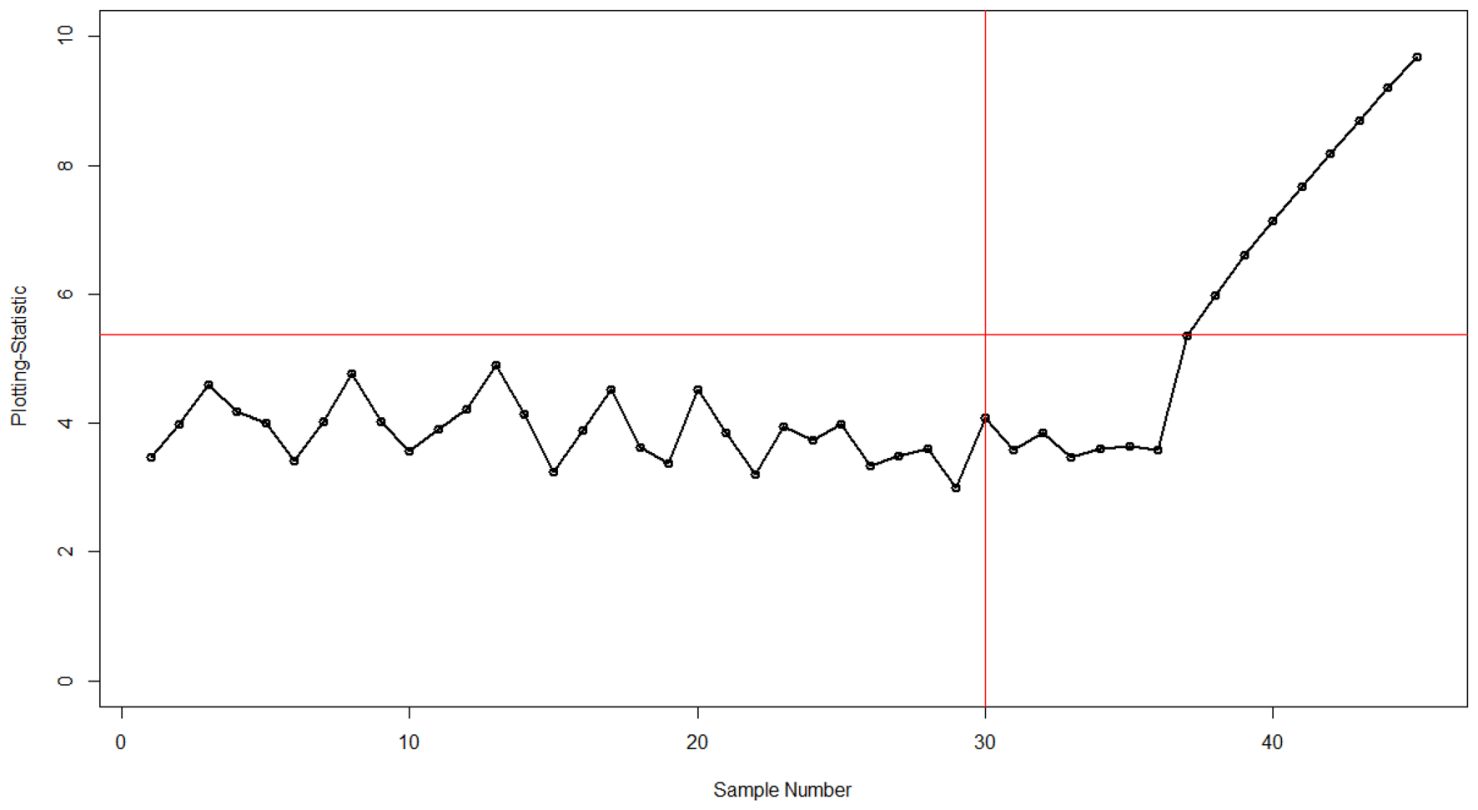
Proposed Adaptive Bayesian Max-EWMA under SELF with $\lambda$ = 0.20.

Table 7 shows the values of phases I and II and the application of existing and proposed charts. It shows the process remains in control for the first 30 values. In phase II, for testing purposes, impurities in the process are introduced by a shift magnitude of 1.00 (${\Delta }_{{\text{X}}}$ = 1.00). The Bayesian MaxEWMA under the SELF method detects this shift at the 40th sampling unit which is also evident from Fig. 1. On the other hand, our proposed Adaptive Bayesian Max-EWMA control chart which incorporates the adaptive values for smoothing constant detects this change in process at an earlier stage. It gives out of control signal at the 37th sample unit. These results are presented in Table 7. The visual illustration of both charts is presented in Figs. 1 and 2. It clearly shows that the proposed chart has a better capability of monitoring the process changes, this early detection shows that the process monitoring has improved and the proposed control chart is quicker at capturing the shifted behavior of the process. It highlights that our integration of the adaptive approach has enhanced the speed and sensitivity of the control chart in detecting shifts in the process mean and variance.

## Conclusion

In this study, we focus on a comprehensive investigation of the adaptive approach and Bayesian methodology to enhance joint process monitoring. We introduce a novel Adaptive Bayesian Max-EWMA control chart that seamlessly integrates an adaptive approach within the Bayesian framework. It utilizes prior and posterior distributions under the SLEF and LLF loss functions. This method is further enhanced with an adaptive approach. The effectiveness of the proposed chart was evaluated through rigorous simulations. Our meticulous analysis, encompassing various parameters, unequivocally demonstrates that the Adaptive Bayesian Max-EWMA chart consistently outperforms its conventional counterpart. To reinforce our conclusions, we conducted a real-life case study. This practical application not only supports the chart's exceptional performance but also provides tangible evidence of its effectiveness in real-world scenarios. The proposed chart's key contributions are as follows: it seamlessly integrates an adaptive approach within the Bayesian framework, allowing it to learn from incoming data and adjust its charting parameters accordingly. It enhances sensitivity to both mean and variance shifts, making it a valuable tool for detecting a wide range of process disturbances. It exhibits superior adaptability to changing process conditions, ensuring its effectiveness even in non-stationary environments. The proposed chart can be applied across a wide range of industries. By providing early warnings of potential process problems, the chart can help reduce scrap, rework, and warranty costs, ultimately improving product quality and customer satisfaction. Future research directions could include investigating the chart's performance under more complex process dynamics, such as non-linear shifts and autocorrelated data. Researchers may also explore the development of adaptive Bayesian control charts for monitoring other process parameters or integrating the chart with multivariate or high-dimensional data.

## Data Availability

The datasets used and/or analyzed during the current study are available from the corresponding author on reasonable request.
